# Relationship of Dentistry to Health Conservation*An address delivered at the twelfth annual meeting of the Association of Life Insurance Presidents in New York City, on December 6, 1918.

**Published:** 1919-02

**Authors:** Harvey J. Burkhardt


### RELATIONSHIP OF DENTISTRY TO HEALTH CONSERVATION *

BY HARVEY J. BURKHARDT, D.D.S.

It is only within a very few years that any considerable number of people have appreciated the value of clean mouths and good teeth. The services of a dentist were sought for the purpose of bringing relief in acute toothache, the repair of broken teeth, or providing artificial substitutes. Not many people have visited a dentist because of an appreciation of the fact that the mouth is a breeding place for many different germs of disease, and therefore should be kept in a hygienic condition. A woeful ignorance exists even today of the far-reaching effect upon the general health of pathological conditions about the mouth. Medical examiners for life insurance companies, industrial establishments, etc., pay particular attention to unusual conditions in every part of the body except the mouth. Small boils and pimples are usually scrutinized with great care to determine if there is any condition that might make the applicant a poor risk. There might be a dozen foci of infection in the mouth, pouring their poison germs into the blood stream and the stomach, but little, if any, attention is paid to these.

This lack of recognition of dental lesions is not confined to the laity. The medical profession itself has been slow to realize the importance of proper mouth conditions. There has often been an antagonism between physicians and dentists, and the patient has been made to suffer, on account of a lack of reasonable co-operation, but happily these conditions are fast passing away. Medical men now realize that many cases of chronic diseases, acute diseases and special local diseases come from mouth infections. For example

* An address delivered at the twelfth annual meeting of the Association of Life Insurance Presidents in New York City, on December 6, 1918.

—neuritis, sciatica, acute paralysis, etc. Ulcerated stomach, diseases of the gall bladder, appendicitis, etc., are often caused by local infections. Cases might be cited of rheumatism, neuritis, arthritis, chronic valvular disease of the heart and many others that have been greatly relieved or cured by proper dental attention.

Bacteria may enter the body in various ways, but the most common situation is in the mouth. There are many places about the mouth to harbor germs of infection—carious cavities in the teeth, pyorrhea pockets, abscesses about the roots of the teeth, the tonsils, etc. That disease germs found in the mouth are a prolific source of disturbance in other parts of the body has been conclusively proven by investigations made by Rosenow and other eminent authorities, who have been able to produce, with bacteria cultures from man, similar diseases in animals. Eminent medical practitioners and health authorities are alive to the fact that mouth sanitation offers a prompt and permanent cure for many of the ills of the body.

The X-ray has been of very great value in diagnosing many of the conditions to be found about the mouth and particularly about the roots of the teeth. Medical men have for some time recognized the fact that many disturbances in remote parts of the body have been caused by infected teeth, and that the removal of the cause has resulted in a speedy restoration to normal health, but a correction of dental disturbances has not always been a cure for every other ill. Much harm has come from the insistence by the medical man upon the extraction of all teeth showing a rarefied area in the picture. At the same time considerable damage has been done by dentists in an unreasonable insistence upon saving every tooth. Medical and dental practitioners will in the end accomplish far greater results, and will be rendering a much more valuable service to their patients, if, instead of a blind insistence upon their individual opinions, they will cooperate with each other more cordially and decide upon operative procedures only after reading and interpreting the pictures in connection with the history of each case. By so doing much needless and ruthless extraction of useful and valuable teeth will cease. Of course, it goes without saying that any competent dentist will give the benefit of the doubt to the patient, rather than insist upon the retention of a tooth that is a menace to health. Equally so the medical man should realize the importance of retaining healthy teeth for the purposes for which they are intended.

There are many well-authenticated cases of a complete restoration to health and normal function by people suffering from insanity, and the various nervous disorders, caused in many cases by nerve pressure from unerupted and impacted teeth. It is also well known that many cures are effected, particularly in rheumatism and neuritis, by removing sources of infection about the mouth and teeth.

The value of the work of the dentist is recognized by medical authorities, health officers, school authorities, the United States Government and prominent business men.

Congress has placed its stamp of approval on dental service by passing a law giving dentists equal rank with medical men. The Surgeon-Generals of the Army and Navy are keenly alive to the value of good teeth and have spoken in complimentary terms of the work performed by dental surgeons. And I am very sorry that Surgeon-General Gorgas is not here today, because I would like to pay him the compliment of saying that it was due practically to his efforts alone that dentistry received the recognition that it did at the last session of Congress. No branch of medicine during the present war has done as much in permanent restorations as have dental and oral surgical departments. It is acknowledged that the proportion of soldiers wounded in the head, face and neck is much greater in this war than any other, and the surgeons and dentists were called upon to demonstrate their skill. In many cases the plastic surgeon could do little, but the dental prosthetist was able by the use of orthodontia appliances, splints, bands, etc., to relieve many distressing conditions. The dentist has frequently been called upon to construct appliances around which the plastic surgeon could reshape and stretch the deformed tissues. Many times the patient will come to the dentist after the wound has healed, when further surgical or dental attention is required, so that the patient will not be disfigured for life. The dental surgeon, by his skill in making appliances, in cases of fractured jaws from accidents and by gun shot wounds, the devising of methods to assist the plastic surgeon in facial restoration, has written one of the most brilliant chapters in the medical history of this war.

A long step ahead was taken by the Government when provision was made to appoint dentists members of local draft and advisory boards, and in formulating regulations with reference to the teeth. The Government recognizes the value of good teeth and clean mouths, by requiring dental inspection, ordering the correction of various troubles about the mouth, by restorations of various kinds, the supplying of artificial substitutes where necessary and the furnishing of tooth brushes. To make more efficient this service, a well-organized bureau has been established in the Surgeon-General's office, in charge of a very capable and able executive. Dental equipment of the very latest and most efficient type has been provided and every hospital and camp contains a well-organized dental clinic in charge of competent dental officers, which is in striking contrast with the Spanish-American War when no dentists were employed.

From the very beginning of the war, no profession responded more promptly or generously than the dental profession. Within three months after the first call, examinations for commissions were closed and enough dentists had volunteered to supply an army of 5,000,000.

The making of an army of over 4,000,000 men dentally fit has been a tremendous task, but it has been done. The soldiers have been made comfortable and fit by the removal of impacted teeth, the treatment of abscesses and diseased teeth and gums, the filling of cavities and the removal of various other pathological conditions. While many of these things will be only temporary in character, by far the greatest permanent good will come from the knowledge that has for the first time been brought to many of them that a clean, comfortable and healthy mouth go hand in hand with good health in promoting greater efficiency. The value of education to secure improved dental conditions in the army does not stop there. When the boys return to their families, they will tell them of the need for paying careful attention to their mouths and teeth as one of the most important things to be done to maintain good health. The Government has not only provided the machinery to take care of the present emergency, but it has inaugurated an educational campaign that will be far-reaching in its effect in promoting good health.

Wonderful strides have been made during the past ten years by the dental profession in carrying forward investigations to show the relationship existing between chronic focal infections associated with teeth and their sysytematic effect. The work has received the cordial approval of many eminent medical men. In an address delivered some three years ago, by Dr. Charles H. Mayo, in which he spoke in complimentary terms of the work done by dentists, he closed with the significant statement, "the next great important step in preventive medicine should be taken by the dental profession." Since that time, much original research work has been done by dentists, individually and through research departments financed by dentists themselves, to demonstrate the faith that is in them and to prove the value of preventive dentistry. Health and school authorities have established clinics in many places and carried on an educational propaganda which is showing wonderful results.

In 1916 there were enrolled in the schools of the State of New York 1,962,946 pupils. In the United States there was a total enrollment of 23,209,029. The per capita cost was $39.37, for the education of each child—a total of $914,804,171. Educational authorities are alive to the value of anything that will improve health conditions, and thereby not only increase the efficiency of the child, but also reduce the cost of education, by shortening the time in school. The item of caring for the backward child and the repeater, due to the loss of time on account of toothache and diseases caused by bad mouth conditions, would in money loss for schooling represent an enormous sum. The loss to parents who are deprived of the services of older children for these reasons, would also represent a large sum.

The economic phase of this question, the permanent good that may come to future generations by the education of children from early childhood to the value and necessity of proper attention to their teeth, and health conservation were thoughts uppermost in the mind of Mr. George Eastman, of Rochester, N. Y., when he founded and endowed the Rochester Dental Dispensary.

After much thought and careful investigation as to the needs and value, and the great good that might come to childhood and humanity generally by early and careful attention and education of the child to the value of dental service, this keen, far-seeing business man decided to establish an institution for the benefit of the children of Rochester. Mr. Eastman is no faddist, neither are his charities or benefactions spectacular. They are decided upon because of their merit or the opportunity to render a real service.

Definite rules for treatment have been established, based upon the weekly wage of the family. Before service is rendered, except in emergency cases, an investigation is made by the social service department to verify the statements of the applicants. A nominal charge of 5c is made for each visit, for the reason that the child will be made to feel that the service is worth something, and to place all children on an equal footing, so that they will feel they are not objects of charity.

The building and equipment cost about four hundred thousand dollars, and an endowment of $750,000 has been provided. Conditional upon making this gift were the requirements that twelve of the leading business men should become interested in the institution by serving upon the Board of Management and contributing $1000 each for five years, and that the City of Rochester should agree to appropriate $20,000 a year for five years to pay for the cleaning of the teeth of the children in the public and parochial schools and orphan asylums. Both of these conditions were at once met.

The building is located not far from the business center and convenient of access from all parts of the city by car lines. It is a beautiful, simple structure and contains the most modern dental and hospital equipment that could be obtained. Provision has been made for 63 complete dental operating units, and the hospital is furnished with every facility and convenience for nose and throat work. This institution was not established for the purpose of doing the ordinary dental relief and repair work. The fundamental thought was to so co-ordinate all of its activities that something might be worked out to prove the value of preventive dentistry. I have no hesitation in saying to you that while much good can be done and great suffering relieved by repair and relief work for the adult, the work is of little value in determining what would be proper methods of treatment for children. An age limit of sixteen years has therefore been established.

It is planned through the education of mothers to have them bring their babies to the dispensary as soon as the first tooth erupts, and by follow-up methods to retain the child as patient until the age of sixteen, believing that by this scheme opportunities for experimentation and study will be given that could not be obtained in any other way and finally result in the standardization of filling materials and methods of procedure that will be of tremendous benefit to coming generations. The retention in good condition of the baby teeth, until such time as they are forced out by the second set, exerts a most beneficial influence upon the permanent teeth. The child that is educated to care for his teeth until the age of sixteen can be depended upon to do so later on and barring unusual conditions with a continuance of proper attention to mouth hygiene, will require little subsequent dental attention. The department of orthodontia is one of the important departments in the Dispensary. Not only will the appearance and comfort of the child be greatly improved, but there will result an improvement in speech by widening the arch, and not infrequently have children below normal mentality been greatly helped by the removal of nerve pressure usually found in a crowded jaw.

The removal of tonsils and adenoids, the operations for cleft palate and harelip will be most important departments of the institution. All of these abnormal troubles of childhood will receive the most expert medical and dental attention that is available and Mr. Eastman has not spared time, money or thought to provide a place in which to carry on remedial and research work, which will be not only for the good of the children of Rochester, but to the children everywhere.

The Dispensary was opened for work on October 15, 1917. The following is a statement of the work done during the first year: Tooth treatments, 57,653; root treatments, 24,903; abscess treatments, 11; prophylactic treatments, 23; root fillings, 2,002; silver fillings, 20,168; cement fillings, 7,267; synthetic fillings, 2,766; gutta-percha fillings, 284; nitrate of silver, 654; capped, 198; crowns, 29; inlays, 3; extractions, 7,824; X-ray, 177; orthodontia, 784; number of visits to Dispensary, 49,122; number of patients, 6,143; completed cases, 4,409.

This work was done by licensed dentists—recent graduates. Thirty-three operators are employed now. While the work done consists principally of fillings and ordinary dental operations, it was done with the thought of preventive dentistry always in mind.

The work of cleaning the teeth of the children in the schools is done by squads of licensed dentists and dental hygienists, the latter being trained in the School of Dental Hygienists conducted by the Dispensary. The squads are provided with a portable equipment consisting of a chair, engine, instruments, sterilizer, etc. All of the prophylactic work—the cleaning of the teeth—is done at the various schools and institutions, under careful and strict supervision.

You will be interested to know that in establishing the School for Dental Hygienists, a new avenue of employment was opened for young women. The term of instruction is one year. The salaries paid graduates are much larger than those usually received by young women,—they earn from $15 to $40 per week. It is a field into which could be turned young women who have taken the places of men in war work, but which will be filled by men later on. The purposes of this school are not to educate women to become dental assistants except for the doing of prophylactic work, which the dentist in a full or busy practice finds more or less of a burden. These young women may be used to good advantage as dental assistants, for the purpose of doing this work in the public school clinics and in manufacturing and business establishments where welfare work is carried on. They may also be employed to give talks and lectures on oral hygiene and other health subjects.

The dental operators and hygienists from the Dispensary made the rounds of the schools in Rochester twice last year. The first time the teeth of 33,664 children were cleaned and the second 41,860. After the teeth have been cleaned a survey is made of the mouth, and if other dental work is necessary, or pathological conditions observed, a card is sent to the parents of the child, calling attention to the necessity of at once correcting the trouble. Literature on the value of clean teeth and a clean mouth, printed in English, Italian, Yiddish and Polish, is sent to the parents.

The work of the young women who have been doing prophylactic work has been most satisfactory, not only in Rochester but in many private dental offices. The Government has also placed its stamp of approval upon their work by giving employment to a considerable number to aid and supplement the work of the regular army dentist in hospitals in Washington.

A school lecturer is employed by the Dispensary, who delivers illustrated lantern slide lectures on oral hygiene and other health subjects. Lectures were delivered to 72,000 children last year.

From this recital of the activities of the Rochester Dental Dispensary, you will observe that a considerable educational propaganda is being conducted. This will be necessary in any community, on account of the woeful ignorance of the public to the value of dentistry in the conservation of health. From the very beginning of the work, it has met with the most cordial reception and approval. The school and health authorities are most enthusiastic and have rendered every assistance in launching this enterprise. While dental clinics are in operation in various places, nowhere else is there an attempt being made to clean the teeth of all the children of a municipality twice a year or where any follow-up method has been inaugurated. There has been a noticeable improvement as regards attendance, and many cases could be cited of a considerable reduction in the number of repeaters, because of the correction of dental troubles. The general hygienic conditions have been improved by this work, and tooth brushes have become fashionable in families where there had been none, or where one was used by the whole family. In many cases the education of the child to appreciate the comfort and benefit of a clean mouth has resulted in an improved condition in other directions.

The education of the public to an appreciation of the tremendous advantages that come from a clean mouth and good teeth, should be one of the important functions of organizations like yours.

Any movement that has for its object the improvement of the physical condition of man, naturally increases the efficiency of the individual and tends to prolong life. There is absolutely no question but that proper attention to the mouth and teeth will do these things.

It seems to me that life insurance companies and other national institutions interested in public health, may render a most valuable public service by becoming interested in this subject.

During this war the large majority of the soldiers and sailors have not only by the Government, but by various philanthropic organizations, been instructed in hygiene and sanitation and the value of health conservation, as the same number could not have been taught in any other manner in many years to come. They have been required to keep themselves and everything with which they have to do in as sanitary a condition as possible, because it is good business so far as the Government is concerned. These lessons will appeal to many of them as being most valuable after the war and many communities will wake up and find soldier sanitary officers among their citizens. This is the time to inaugurate a general health campaign. This war has strikingly proven the laxity of not only the health laws but the educational as well, by disclosing the thousands who were inducted into the army who could not sign their names. Hand in hand with business reconstruction should go the reconstruction of the health laws to make healthier and better citizens. The campaign to save babies has resulted in much good, and while there have been great advances by medical men along this line, not so much has been done to prolong adult life. This country is face to face with an industrial situation that will tax the brains of the wisest to bring about a right solution. Europe will no longer furnish the common labor for this country and we will be obliged to furnish our own. Over six per cent. of the accident and sick loss in this country may be cut in half by improving housing, sanitary, hygiene and food conditions.

You will no doubt be surprised to hear that not more than ten per cent. of the inhabitants of this country regularly employ a dentist, or appreciate the value of dental service. In developing a health campaign due consideration should be given to the available number of physicians and dentists. There are not today dentists enough to properly look after the teeth of a sixth of the population of this country. In educating the people to appreciate this service it will also be necessary to increase professional educational facilities, and make the work attractive enough to induce young people to take up professional work. There is no association of which I have any acquaintance that is as well organized or equipped as is yours to lead in movements for the improvement of the health of the people. The advantages to be derived by improved health conditions will not only result in greater happiness and efficiency, but will measurably increase the length of life.

Life insurance can be made more popular by reducing its costs, and more business will be made for companies and for agents. The tremendous army of life insurance solicitors, medical examiners and employees generally, could exert a wonderful influence in every community, because none is without them. They could show municipal, school authorities and philanthropists the value of medical and dental clinics, and exert an influence in advancing the general health of the people, far beyond that of any other organization. What Mr. Eastman has done for Rochester probably will be done by other men of wealth in other places, when they can be shown that their investment will bring a larger measure of comfort and happiness not only to the immediate generation, but the future ones by demonstrating the value of a clean mouth and good teeth, and by devising and discovering methods and agencies that will hasten the day of preventive dentistry.

